# Study protocol: the Whitehall II imaging sub-study

**DOI:** 10.1186/1471-244X-14-159

**Published:** 2014-05-30

**Authors:** Nicola Filippini, Enikő Zsoldos, Rita Haapakoski, Claire E Sexton, Abda Mahmood, Charlotte L Allan, Anya Topiwala, Vyara Valkanova, Eric J Brunner, Martin J Shipley, Edward Auerbach, Steen Moeller, Kâmil Uğurbil, Junqian Xu, Essa Yacoub, Jesper Andersson, Janine Bijsterbosch, Stuart Clare, Ludovica Griffanti, Aaron T Hess, Mark Jenkinson, Karla L Miller, Gholamreza Salimi-Khorshidi, Stamatios N Sotiropoulos, Natalie L Voets, Stephen M Smith, John R Geddes, Archana Singh-Manoux, Clare E Mackay, Mika Kivimäki, Klaus P Ebmeier

**Affiliations:** 1 Department of Psychiatry, University of Oxford, Warneford Hospital, Oxford OX3 7JX, UK; 2 Nuffield Department of Clinical Neurosciences, University of Oxford, Oxford, UK; 3 Department of Epidemiology & Public Health, University College London, London, UK; 4 Center for Magnetic Resonance Research (CMRR), University of Minnesota, Minneapolis, MN, USA; 5 Icahn School of Medicine at Mount Sinai, New York, NY, USA; 6 Centre for Clinical Magnetic Resonance Research, University of Oxford, Oxford, UK; 7 Centre for Research in Epidemiology and Population Health, Hôpital Paul Brousse, INSERM, U1018, 94807 Villejuif, Cedex, France

**Keywords:** Epidemiology, Magnetic resonance imaging, Diffusion tensor imaging, White matter, Functional MRI, Connectome, Resting state brain networks, Neuropsychology, Dementia, Affective disorders

## Abstract

**Background:**

The Whitehall II (WHII) study of British civil servants provides a unique source of longitudinal data to investigate key factors hypothesized to affect brain health and cognitive ageing. This paper introduces the multi-modal magnetic resonance imaging (MRI) protocol and cognitive assessment designed to investigate brain health in a random sample of 800 members of the WHII study.

**Methods/design:**

A total of 6035 civil servants participated in the WHII Phase 11 clinical examination in 2012–2013. A random sample of these participants was included in a sub-study comprising an MRI brain scan, a detailed clinical and cognitive assessment, and collection of blood and buccal mucosal samples for the characterisation of immune function and associated measures. Data collection for this sub-study started in 2012 and will be completed by 2016. The participants, for whom social and health records have been collected since 1985, were between 60–85 years of age at the time the MRI study started. Here, we describe the pre-specified clinical and cognitive assessment protocols, the state-of-the-art MRI sequences and latest pipelines for analyses of this sub-study.

**Discussion:**

The integration of cutting-edge MRI techniques, clinical and cognitive tests in combination with retrospective data on social, behavioural and biological variables during the preceding 25 years from a well-established longitudinal epidemiological study (WHII cohort) will provide a unique opportunity to examine brain structure and function in relation to age-related diseases and the modifiable and non-modifiable factors affecting resilience against and vulnerability to adverse brain changes.

## Background

Over the next few decades, increases in life expectancy will result in fundamental changes to the population structure. Associated with this demographic change, health and social care services will need to cope with a greater prevalence of mental and neurological disorders. Clinical depression and cognitive decline have a combined estimated prevalence of 7-20% in the population over 65 years [1-4]. Furthermore, according to some estimates, the number of people with neurodegenerative disorders will quadruple in the next 20 years causing a significant increase in the cost of care [5]. To extend the productive period in citizens’ lives and to reduce costs of care in late life a greater knowledge of prevention and treatment of these common conditions is needed. This will not be possible without a better understanding of the causal mechanisms of disease and, equally importantly, the factors associated with resilience to age-related dysfunction [6].

The Whitehall II (WHII) study of 10,308 British civil servants provides a remarkable source of longitudinal data to explore factors hypothesized to affect brain health and cognitive ageing (Table 1). The cohort was established in 1985 at University College London (UCL) with the aim of advancing knowledge of the causal chain through which social circumstances influence health [7]. By September 2011, the study had acquired 25 years of rich social, behavioural and biological data enabling its transformation into a unique study of ageing. Of the original 10,308 non-industrial civil servants recruited in Phase 1, 6035 participated in the ‘Phase 11’ assessment in 2012–13. As participants in this study continue into older adult life, the research now focuses on life course factors affecting health and personal functioning at older ages.

**Table 1:** Phases of Whitehall II with available measures

	***Phase 1 1985-88***	***Phase 3 91-93***	***Phase 5 97-99***	***Phase 7 03-04***	***Phase 9 07-09***	***Phase 11 12-13***
***Ages [years]***	35-55	39-64	45-69	50-74	55-79	60-85
***Participants [n]***	10308	8637	7830	6967	6755	6035
***Social circumstances & behaviour, smoking, alcohol, exercise, sleep diet***	×	×	×	×	×	×
***Biological measures: blood pressure, BMI, lipids, glucose, insulin, stored blood (−80C)***	×	×	×	×	×	×
***2-h oral glucose tolerance test***	--	×	×	×	×	×
***Inflammatory markers***	--	×	×	×	×	--
***Autonomic (HRV)***	--	--	×	×	×	×
***Genetic material***	--	--	--	×	×	×
***Psychosocial factors: work, social support, − participation, care provision***	×	×	×	×	×	×
***Health outcomes: CHD, stroke, diabetes, cancer, mortality, medications***	×	×	×	×	×	×
***Function: physical, social & mental***	×	×	×	×	×	×
***Cognitive tests, physical/lung function tests***	--	--	×	×	×	×

× = present; -- = absent.

We randomly selected 800 WHII Phase 11 participants to take part in the WHII imaging sub-study, which includes a detailed clinical and cognitive assessment, measurement of immune parameters and a magnetic resonance imaging (MRI) scan. MRI scans provide a non-invasive window into the living brain, giving unique access to understanding normal and pathological processes that affect brain structure and function. Incorporating state-of-the-art imaging techniques and cognitive measures with the WHII’s longitudinal dataset of social, behavioural and biological variables, represents a unique opportunity to study the ageing process and to directly link 25-year exposure history to old-age cognition and a variety of measures of ‘brain health’. Analysis of immunological variables and linkage of these studies with behavioural and imaging data enables a more comprehensive investigation of the pathophysiological processes of dysfunction and cognitive impairment in later life.

In this paper, we provide a description of the study’s organisation and funding structure, its participants’ inclusion/exclusion criteria and of the cognitive, imaging and blood specimen protocols employed in the study. For the imaging protocol, careful consideration has been given to harnessing the most recent technical developments, whilst maintaining clinical relevance. A preliminary description of the techniques to be used to pre-process and examine MRI-related measures will also be presented, and results of a direct comparison between a recently developed and a more standard MRI acquisition approach for investigating brain functional organization will also be shown. We provide an overview of our original hypotheses at the time of application for funding.

## Methods/design

### Study organization and funding

The sub-study is funded by the Lifelong Health and Wellbeing Phase-3 programme grant “Predicting MRI abnormalities with longitudinal data of the Whitehall II sub-study” (MRC-G1001354; Ebmeier KP (PI), Geddes JR, Kivimäki M, Mackay CE, Singh-Manoux A, Smith SM), as well as the HDH Wills 1965 (English Charity Register: 1117747; Ebmeier KP (PI)), and the Gordon Edward Small Charitable (Scottish Charity Register: SC008962; Ebmeier KP (PI)) Trusts. Collection of blood and buccal mucosal samples for a characterisation of immune function and associated measures is funded by the UK Medical Research Council programme grant K013351 (“Adult determinants of late life depression, cognitive decline and physical functioning - The Whitehall II Ageing Study”, Kivimäki M (PI), Singh-Manoux A, Brunner E, Batty GD, Kumari M, Ebmeier KP, Hingorani A) and the ESRC professional fellowship scheme to Kivimäki.

Ethical approval was granted generically for the “Protocol for non-invasive magnetic resonance investigations in healthy volunteers” (MSD/IDREC/2010/P17.2) by the *University of Oxford Central University / Medical Science Division Interdisciplinary Research Ethics Committee* (CUREC/MSD-IDREC), who also approved the specific protocol: “Predicting MRI abnormalities with longitudinal data of the Whitehall II sub-study” (MSD-IDREC-C1-2011-71). The *Health Research Authority NRES Committee South Central – Oxford B* approved the Study: “The Whitehall II Immune Function Sub-study” (REC reference: 13/SC/0072, IRAS project ID: 120516).

The study follows the Medical Research Council (MRC) Policy on data sharing, i.e. images and other data will be available for analysis by other groups after completion of the study, as is the case with the Whitehall II study (see http://www.ucl.ac.uk/whitehallII/data-sharing[8,9].

### Participants’ recruitment and cognitive protocol description

#### Participants

**Figure 1:**
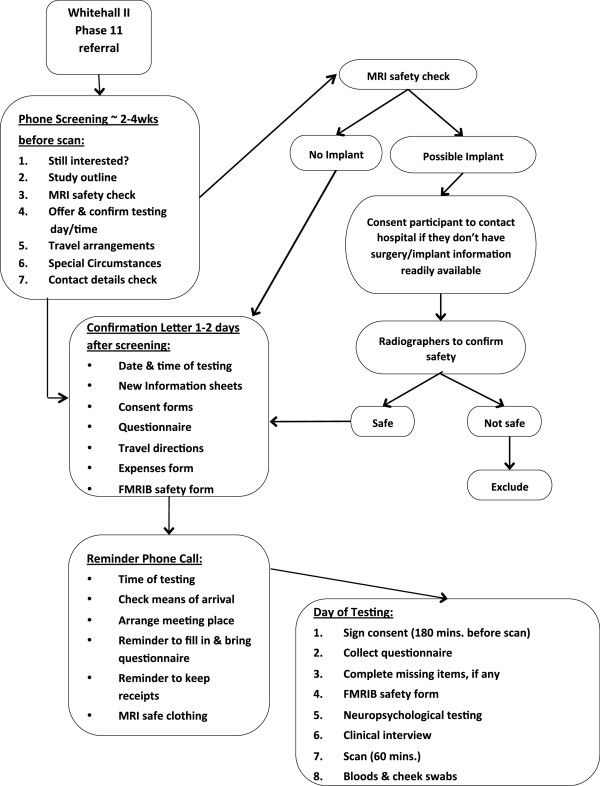
Flow-chart summarising the stages of the WHII imaging sub-study.

In order to make the sample as representative as possible of the cohort at baseline, a random sample of 800 WHII Phase 11 participants willing and able to give informed consent have been invited to attend the imaging sub-study at the Oxford Centre for Functional MRI of the Brain (FMRIB). To achieve a sufficient number of participants with depression, we added 30 participants with depressive symptoms based on previous WHII clinical examinations. We excluded participants with contraindications to MRI scanning (including but not limited to a history of claustrophobia, certain metallic implants and metallic injury to the eye) or who were unable to travel to Oxford without assistance. A schematic flow-chart describing the different stages of the study is provided in Figure 1.

### Clinical and cognitive assessment

Each participant recruited for the WHII imaging sub-study undergoes a detailed clinical and cognitive assessment lasting up to two hours.

The clinical assessment consists of a (A) self-administered questionnaire, a (B) semi-structured clinical interview and (C) cognitive assessment (median = 56 minutes, interquartile range: 51–61 minutes).

#### Self-administered questionnaire

*General Health Questionnaire-30 (GHQ-30;*[10]*)*: The GHQ-30 is a 30-item self-administered screening questionnaire for the detection of psychiatric illness that accompanies ill-health, in non-psychiatric clinical and community settings (routinely applied from scan 200).

*Mood Disorder Questionnaire (MDQ;*[11]*)*: The MDQ is a brief self-report questionnaire for the assessment of life time history of bipolar disorders, based on the DSM-IV.

*Centre for Epidemiological Studies Depression Scale (CES-D;*[12]*)*: The CES-D is short self-report scale that measures major depressive symptomatology in the general population.

*State and Trait Anxiety Inventory (STAI;*[13]*)*: The STAI measures both S (state)- and T (trait)-Anxiety in clinical and research settings [14]. It is a self-administered questionnaire that consists of twenty statements assessing how the individual feels at the moment (S-Anxiety) and twenty assessing how they generally feel (administered to n = 15 before routinely applied from scan 200).

*CHAMPS Physical Activity Questionnaire for Older Adults*[15]: The CHAMPS is a self-administered physical activity questionnaire for older persons. Participants report the weekly frequency and duration of various physical activities, typically undertaken by older adults, allowing calculation of metabolic equivalent of task (MET) and caloric expenditure values per week.

*Locus for Causality Exercise Questionnaire (LCE;*[16]*)*: The LCE is a 3-item self-administered scale that assesses how much an individual feels that they choose to exercise (internal perceived locus of causality) rather than have to exercise for some reason (external perceived locus of causality). It is thought that individuals are more likely to engage in physical exercise when the perceived locus of causality is internal [17].

*Pittsburgh Sleep Quality Index (PSQI;*[18]*)*: The PSQI is a self-rated questionnaire made up of seven component scores that assess sleep quality and disturbance over a one month period in clinical and research settings.

*Jenkins Sleep Questionnaire (JSQ;*[19]*)*: The JSQ is a 4-item self-rated questionnaire for the assessment of sleep disturbances over a month period.

*Life-Orientation Revised (LOT-R;*[20]*)*: The LOT-R was devised to measure individual optimism for future events in the general population.

*Life Events*[21,22]*)*: A modified version of the List of Threatening Experiences questionnaire (LTE-Q) is used, in which participants are asked about seven types of stressful life events. Participants are asked to remember if any of the events happened to them in the past, and when they happened.

*MacArthur stress reactivity questionnaire*[23]: It is a nine-item self-rated questionnaire in which the participant is required to rate nine statements on a 5-point scale, regarding to how they handle their emotions in stressful situations.

*Penn State Worry Questionnaire Ultra-brief Version (PSWQ;*[24]*)*: The PSWQ ultra-brief is the 3-item version of the widely used self-report questionnaire for pathological worry, the 16-item long PSWQ. The 3-items capture pathological worry as defined by the DSM-IV; perceived uncontrollability, multiple domains and high frequency of worry. The PSWQ was introduced into the assessment after scan 200.

*Handedness (*[25]*)*: It is a self-administered questionnaire that assesses which is the participant’s preferred hand to complete a list of twelve tasks, as well as left-handedness in the family.

Participants also provide information on medical history (detailing hospitalizations, longstanding illnesses, diseases or medical conditions), alcohol and nicotine intake, and general information, such as age and education. Their blood pressure is measured twice in a sitting position, after the cognitive protocol (OMRON HEM-907; OMRON Healthcare UK Ltd., Milton Keynes).

#### Semi-structured clinical interview

*Structured Clinical Interview for DSM-IV-TR Axis I Disorders*[26]: The SCID-I is a semi-structured interview for diagnosing current and past DSM-IV Axis I disorders and is administered by a trained graduate psychologist or psychiatrist.

#### Further structured assessments

Further tests were performed when the clinical history and SCID data of the participant suggested a diagnosis and indicated that a more detailed assessment was required (the number of each test carried out so far is listed in brackets):

*Hamilton Depression Scale (HAMD;*[27]*)*: The HAMD is a 17-item severity scale administered to individuals diagnosed with ‘affective disorder of depressive type’. It has been devised to quantify the intensity of the depressive symptoms of the patient, based on the necessary information elicited by the interviewer. This scale was administered to n = 16 participants currently symptomatic on the SCID-I [26].

*Young Mania Rating Scale (YMRS;*[28]*)*: The YMRS is an 11-item rating scale for the assessment of manic symptoms based on the subjective report of the patient’s experience over the past forty-eight hours. It follows a rating style of symptom severity similar to that of the HAMD [27] and is administered to participants currently symptomatic on the SCID-I [26] (n = 2).

*Yale-Brown Obsessive Compulsive Scale (Y-BOCS;*[29,30]*)*: The Y-BOCS is a clinician-rated ten-item scale of the severity of symptoms of obsessive-compulsive disorder, with separate subtotals for obsessions and compulsions. This scale is applied to participants currently symptomatic on the SCID-I [26] (n = 0).

*CAGE Questionnaire*[31]: Four questions make up this questionnaire to detect dependence on alcohol. They request information on whether the individual needs to “**C**ut down” their drinking, feels **A**nnoyed by criticism of their drinking, feels **G**uilty about their alcohol use, and whether they use alcohol first thing in the morning as an ‘**E**ye-opener’. This scale is administered to participants currently symptomatic on the SCID-I [26] (n = 6).

*Brief Psychiatric Rating Scale (BPRS;*[32]*):* The BPRS is an 18-24-point rating scale for the assessment of psychotic symptoms, and is used in both clinical and research settings. This scale is administered to participants currently symptomatic on the SCID-I [26] (n = 0).

#### Cognitive assessment

Cognitive test battery administered to all participants:

*Montreal Cognitive Assessment (MoCA;*[33]*)*: The MoCA is a 30-point cognitive screening test assessing multiple cognitive domains: a) visuo-spatial abilities (4 points), assessed using a three-dimensional cube-drawing (1 point) and a clock-drawing task (3 points); b) short-term memory recall task (5 points), which involves learning 5 nouns and recalling them approximately 5 minutes afterwards; c) executive function (3 points), which include an alternation task (1 point) and a verbal abstraction task (2 points); d) attention, orientation and working memory (6 points), which are evaluated using a forward- and backward-digit task (2 points), a sustained attention task (1 point), and a serial subtraction task (3 points); e) language (6 points), which is measured using a three-item naming task (3 points), the repetition of two syntactically complex sentences (2 points) and a phonemic fluency task (1 point); and f) orientation to time and space (6 points). Participants receive an additional (1 point) if their education level is ≤ 12 years. Since the MoCA assesses multiple cognitive domains, it is a useful cognitive screening tool for several neurological diseases, such as Parkinson’s disease, vascular cognitive impairment, Huntington’s disease, multiple sclerosis, and other conditions, such as traumatic brain injury, depression and schizophrenia [33,34].

*Trail Making Test (TMT) versions A and B* ([35,36]: The TMT is a visual attention and task-switching test consisting of two parts in which the subject is instructed to connect a set of twenty-five consecutive dots (A: numbers and B: numbers and letters) on a sheet of paper as fast as possible while still maintaining accuracy. It provides information about visual search speed, speed of processing, mental flexibility, as well as executive functioning [36]. It is sensitive to the detection of cognitive impairment including Alzheimer’s disease [37].

*Rey Complex Figure Test and Recognition Trial (RCFT;*[38,39]): The RCF involves copying and then recalling a complex geometric diagram at increasing time intervals [40]. Different cognitive abilities are needed for a correct performance, including visuo-spatial abilities, memory, attention, planning, and working memory. It is used to investigate the effects of brain injury and to test the presence of neurodegenerative conditions [41].

*Verbal fluency test (adapted from the Addenbrooke’s Cognitive Examination Revised (ACE-III)*[42]*)*: The verbal fluency test requires participants to say as many words as possible from a category (animals) in a specified time (60 seconds). It is used to investigate the presence of cognitive impairment, neurodegenerative and psychiatric disorders [43].

*Hopkins Verbal Learning Test-Revised (HVLT-R;*[44]*)*: The HVLT-R test provides a measure of verbal learning and memory ability [45,46]. The participant is required to learn a list of twelve words over the course of three trials, and recall and recognise them at increasing time intervals. It is widely used to test the presence of amnestic disorders [47,48].

*Boston Naming Test (BNT-60;*[49]*)*: The BNT-60 is a 60-item test graded in difficulty used to measure semantic memory ability and requires naming of a series of images shown to the participant [50]. It is used in individuals with aphasia or any language disturbance caused by neurological insults, such as stroke or neurodegenerative disorders [51].

*Digit Span (DS) and Coding (DC) tests from the Wechsler Adult Intelligence Scale - Fourth Edition (WAIS-IV;*[52]*)*: The DS test is used to investigate short-term memory abilities. It includes recall of a lengthening list of digits forwards, backwards, and rearranged in ascending sequence (DSF, DSB, DSS) [53,54]. In the DC test participants have to write the appropriate novel symbol for each number within a given time.

*Test of Premorbid Functioning (TOPF;*[55]*)*: The TOPF consists of a list of seventy written words, which must be read aloud and is marked according to pronunciation. The TOPF is used to estimate an individual's level of intellectual functioning before the onset of injury or illness. Premorbid IQ can be calculated from the raw score, adjusted for sex and years of education.

*Dots and letters (adapted from the Addenbrooke’s Cognitive Examination III;*[42]*)*: The participant is asked to count four sets of dots without pointing to them and identify four partially drawn letters. These tasks assess perceptual abilities.

*CLOX*[56]: The CLOX is a clock drawing task; in the first part the participant is given a set of instructions to draw a clock and in the second part the examiner draws a clock face, which the participant then has to copy. The CLOX was designed to assess executive impairment and non-executive failure, and is used to discriminate dementia sub-groups [56].

*Cambridge Neuropsychological Test Automated Battery Reaction Time touchscreen version (CANTAB RTI; CANTABeclipse 5.0; Cambridge Cognition Ltd.*http://www.camcog.com*)*: The CANTAB RTI is a computerised (touchscreen) latency task that measures latency and movement time without having to control for tremor. The task is divided into a simple and 5-choice reaction time stage. During the task the participant must react as soon as a yellow dot appears; moving their finger on the screen from a pre-defined location to the location of the yellow dot. In the simple stage the yellow dot always appears in the same location, and in the five-choice stage in one out of five potential locations. The CANTAB RTI is often used to assess visuo-spatial and visuo-motor coordination abilities [57], motor speed [58], and understand sustained attention and reaction time [59].

*Purdue Pegboard Test*[60,61]: The Purdue Pegboard measures two types of dexterity; gross movement of the fingers, hands and arms, and fine fingertip dexterity. The participant places pins into a row of holes using right, left and both hands (gross movement) and assembles a set of structures from pins, collars and washers using both hands (fine dexterity) as fast as they can, within a given time. The Purdue Pegboard test was devised for employee selection for industrial jobs but is also used in clinical settings. Impaired peg placement was found among patients with Parkinson’s disease [62,63]), Huntington’s disease [62] and schizophrenia [64]. The Purdue Pegboard can also aid lateralization of function [65]. Healthy older people without neuropsychiatric or other disease who showed MRI white matter hyperintensities (WMH) performed worse on the assembly subtest (fine dexterity) than those without WMH [66].

### Imaging protocol description

Scanning is carried out at the Oxford Centre for Functional MRI of the Brain (FMRIB) using a 3 T Siemens Magnetom Verio (Erlangen, Germany) Scanner with a 32-channel receive head coil. The neuroimaging protocol comprises both structural and functional sequences and lasts approximately 50 minutes. MRI sequences include: a) high-resolution T1-weighted, b) diffusion MRI (dMRI), c) resting-state functional MRI (rfMRI), d) Fluid Attenuated Inversion Recovery (FLAIR) and e) T2*. A full description of the MRI parameters adopted in our sequences is provided in Table 2.

**Table 2:** MRI sequences and parameters used in the study; S and T define Sagittal and Transversal orientation, respectively

	**Structural**				**Functional**	
**Sequence**	MEMPR	FLAIR	T2*	dMRI	Multiband	Standard
**Condition**	————————————	————————————	———————————	————————————	Resting	Resting
**TR in ms**	2530	9000	36	8900	1300	3000
**TE in ms**	1.79/3.65/5.51/7.37	73	30	91.2	40	30
**Flip angle**	7°	150°	15°	————————————	66°	90°
**Voxel in mm**^**3**^	1x1x1	0.9x0.9x3	0.7x0.7x1.5	2x2x2	2x2x2	3x3x3
**FoV read**	256	220	220	192	212	192
**FoV phase**	100%	100%	81.3%	100%	100%	100%
**Base resolution**	256	256	320	96	106	64
**Phase resolution**	100%	100%	100%	100%	100%	100%
**TI in ms**	1380	2500	———————————	————————————	————————————	————————————
**Bandwidth**	651 Hz/Px	283 Hz/Px	170 Hz/Px	1680 Hz/Px	1814 Hz/Px	2368 Hz/Px
**Orientation**	S	T	T	T	T	T
**b-value**	—————————	———————————	——————————	1500 s/mm^2^	———————————	—————————————
**N. of volumes**	—————————	———————————	—————————	——————————	460	200
**N. of directions**	——————————	———————————	——————————	60 + 5 b = 0 s	————————————	—————————————
**Acquisition time**	6 m 12 s	4 m 14 s	4 m 17 s	9 m 56 s	10 m 10 s	10 m

### T1-weighted

This sequence is primarily used to study grey matter (GM) structural macroscopic tissue in both cortical and subcortical brain regions. GM reductions have been widely associated with impending disease and age-related cognitive dysfunction [67-69].

A Multi-Echo MPRAGE (MEMPR) with motion correction, developed at the Massachusetts General Hospital (MGH, Boston), was employed [70,71]. This sequence has the advantage of combining the properties of the classical MPRAGE sequence, which has high contrast aiding cortical segmentation, with Multi-Echo FLASH, which improves segmentation of subcortical regions.

**Figure 2:**
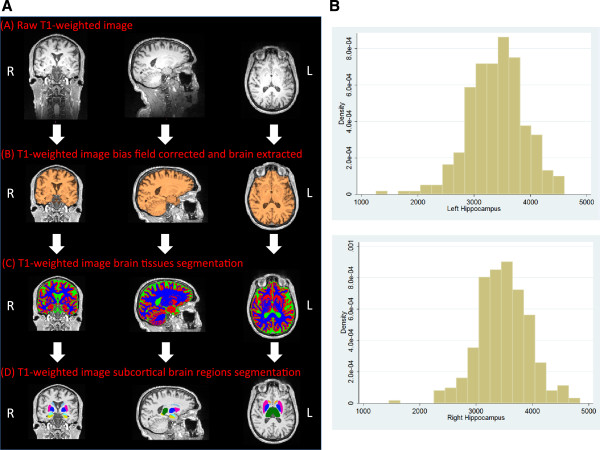
**T1-weighted imaging – analysis pipeline and initial volumetric results. A**. Schematic figure representing the pipeline set up to analyse T1-weighted images. The **(A)** raw T1-weighted image is initially **(B)** pre-processed (bias field corrected, neck cropped and brain extracted). Subsequently, **(C)** brain tissues are segmented into gray matter (GM, red), white matter (WM, blue) and cerebrospinal fluid (CSF, green). Finally, **(D)** subcortical brain regions are segmented. R and L define the right and the left hemisphere, respectively. **B**. Histogram of hippocampal sizes in the first 300 participants. Volume measures in 10^-3^ ml.

The pre-processing pipeline includes: a) re-orientating images to the standard (MNI) template, b) bias field correction, c) registration to the MNI template using both linear (FLIRT) and non-linear (FNIRT) registration tools and d) brain extraction. Brain tissues are segmented using FMRIB's Automated Segmentation Tool (FAST) that allows extracting measures of total GM, WM and cerebrospinal fluid (CSF). FIRST (http://fsl.fmrib.ox.ac.uk/fsl/fslwiki/FIRST) [72], an automated model-based segmentation/registration tool, is applied to extract subcortical grey matter structures. Brain tissues and subcortical regions are visually inspected to ensure an accurate segmentation (Figure 2A,B).

### Diffusion MRI (dMRI)

Diffusion MRI exploits the principles of traditional MRI to measure the random motion of water molecules and subsequently to 1) infer information about white matter (WM) microstructural properties and 2) delineate the gross axonal organisation of the brain [73]. WM is characterised by bundles of myelinated axons surrounded by myelin sheaths that are built up by layers of membranes. This restricts diffusion of free water molecules; i.e. the myelin layers and the axonal membrane cause a lower restriction along than across the axon and thus a higher anisotropy.

A number of strategies were used to minimise distortions caused by, for example, magnetic susceptibility, eddy-currents, and subject-motion. We employed monopolar diffusion encoding gradients with parallel imaging (GRAPPA) to minimise echo time, which increases the signal to noise ratio (SNR), at the cost of a small increase in eddy-current distortion. We used a recently developed dMRI correction strategy that takes advantage of the complementary information from pairs of diffusion images acquired with reversed phase-encoding (PE) directions to correct for susceptibility-induced distortions [74]. A single non-diffusion weighted (b-value = 0 s/mm^2^) volume with reversed PE was combined with the non-reversed dMRI data to estimate an off-resonance field, which is then applied to correct susceptibility distortions [75].

**Figure 3:**
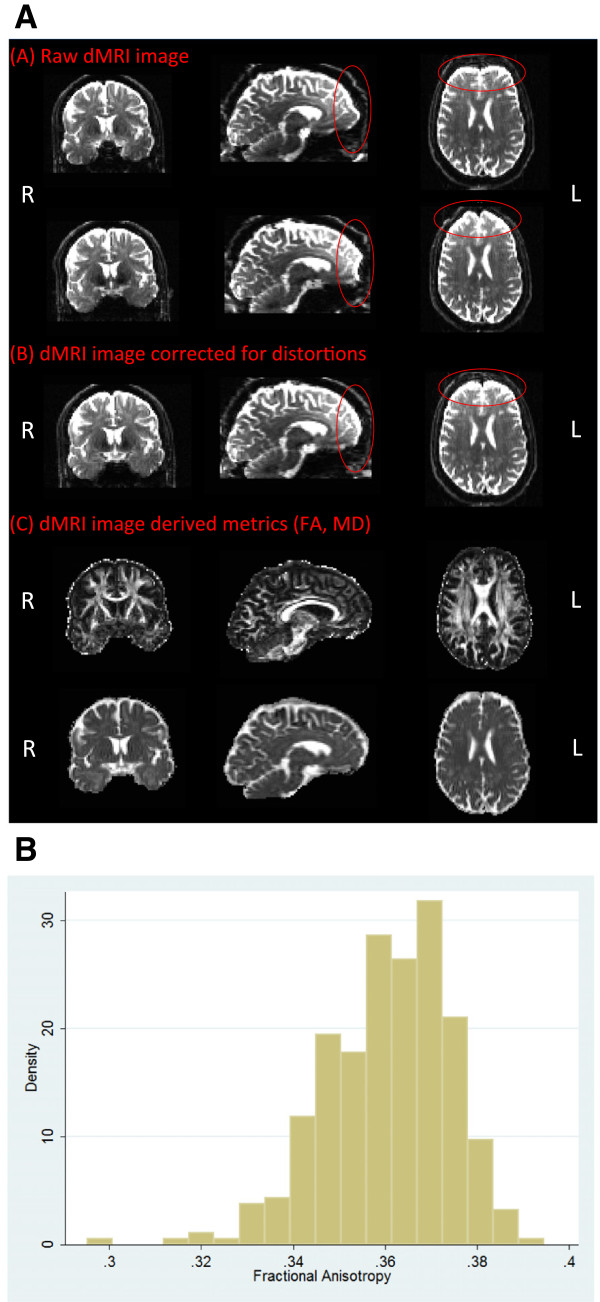
**Diffusion MRI - analysis pipeline and initial results for fractional anisotropy. A**. Schematic figure representing the raw data (b=0) for both anterior-to-posterior and posterior-to-anterior encoding directions **(A)** and after application of the two reversed phase encoding **(B)**. Red circles show before and after correction for distortions. Diffusion MRI (dMRI)-derived metrics for fractional anisotropy (FA) and mean diffusivity (MD) are also shown **(C)**. R and L define the right and the left hemisphere, respectively. **B**. Histogram of mean fractional anisotropy in the first 300 participants.

The Pre-processing pipeline uses a recently developed approach that simultaneously considers and corrects for susceptibility-induced distortions, eddy-currents and head motion (based on methods developed and applied to the Human Connectome Project (HCP) diffusion MRI data [74]). Briefly, a generative model approach is used to estimate all types of distortion and a single resampling step with spline interpolation is used to correct for all of them simultaneously. Fractional anisotropy (FA), mean diffusivity (MD), axial diffusivity (AD) and radial diffusivity (RD) maps are generated using DTIFit, part of FMRIB’s Diffusion Toolbox (http://fsl.fmrib.ox.ac.uk/fsl/fdt), that fits a diffusion tensor model at each voxel [76,77] (Figure 3, b). Subsequently, crossing fibre orientations can be estimated, and probabilistic tractography can be performed to reconstruct white matter bundles and assess structural connectivity [78].

### Resting-state functional MRI (rfMRI)

rfMRI is used to investigate resting state networks (RSNs), which comprise brain regions sharing a common time-course of spontaneous fluctuations, and are thought to represent functionally-critical neuronal networks that reflect properties of functional brain organisation [79]. RSNs have been consistently observed across subjects, sessions and functional brain imaging modalities (fMRI, PET, EEG), and their presence has also been reported in studies when participants were asleep, and in anaesthetized monkeys and rats [79-85]. Although potentially harder to interpret than task-based fMRI, the rfMRI approach has the considerable advantage of providing an assay of brain function without requiring subjects to undertake a specific task, particularly in cases where a subject may be less able to understand and/or respond to complex instructions.

We compared a recently developed Multiband MRI sequence [86,87] with ‘standard’ EPI. Multiband provides a considerable improvement in temporal (Multiband: 1.3 seconds vs. Standard EPI: 3 seconds) and spatial (Multiband: 2 mm isotropic vs. Standard EPI: 3 mm isotropic) resolution, which allows: a) better definition of the spatial maps, b) wider frequency range exploration in time-series analyses and c) more detailed network analyses. To ensure that the new multiband sequence was robust in our older population, we acquired both sequences (standard and multiband) on a subset of participants (N = 76). Results of this comparison will be presented in the next section. In all cases subjects were instructed to lie in dimmed light with their eyes open, blink normally, but not to fall asleep.

rfMRI data pre-processing (motion correction, brain extraction, high-pass temporal filtering with a cut-off of 100 s, and field-map correction is carried out using MELODIC (Multivariate Exploratory Linear Optimized Decomposition into Independent Components, part of FSL http://fsl.fmrib.ox.ac.uk/fsl/fslwiki/melodic/) [88,89]. In order to reduce the presence of spatially and/or temporally-related artefacts a data-cleaning approach is applied. Single-subject independent component analysis (ICA) is followed by an automatic component classification with FMRIB's ICA-based X-noiseifier (FIX) to identify and regress out the “signal” of the artefactual components reflecting non-neuronal fluctuations [90,91]. The pre-processed and “cleaned” functional data are registered to the individual's structural scan and standard space images using FNIRT, then optimized using boundary-based-registration approach [92], and finally spatially smoothed using an isotropic Gaussian kernel of 6 mm full width at half maximum (FWHM).

**Figure 4:**
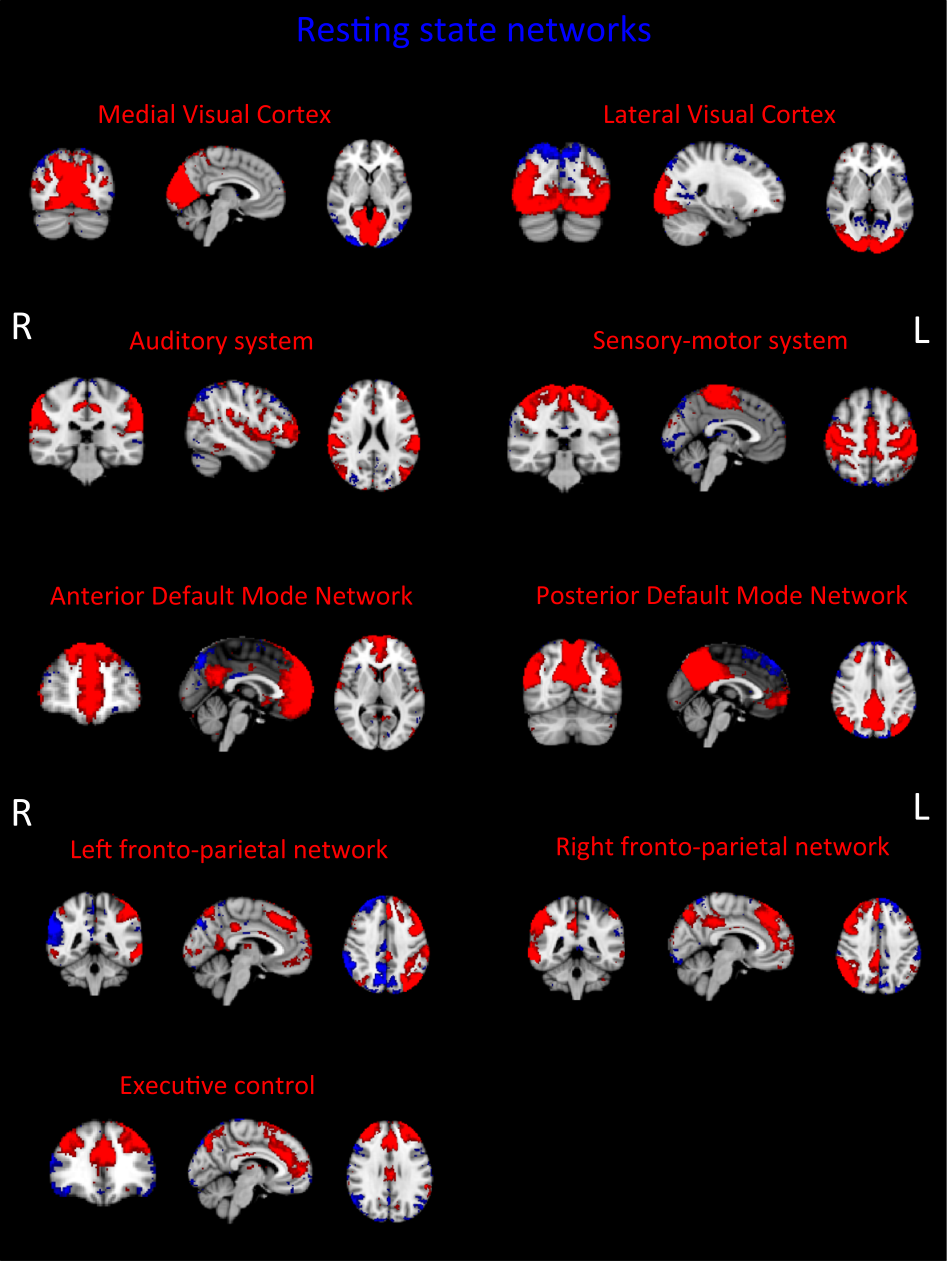
**Resting state networks (RSNs) derived from Multiband data.** Images represent group maps of 50 subjects. All RSN maps are thresholded at Z = 3. R and L define respectively the right and the left hemisphere. Maps reported here are not smoothed.

**Figure 5:**
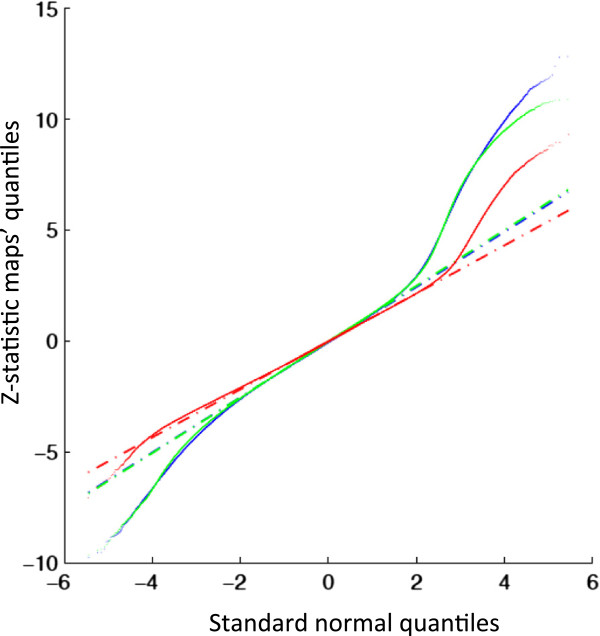
**Q-Q plots of group maps’ z values.** Continuous lines represent Q-Q plots for Multiband (green), Standard EPI (red) and Human Connectome Project template (blue). Dashed-dotted lines join the first and third quartiles of each distribution, to help evaluate the linearity of the data.

This project represents the first major application of the Multiband sequence in older adults: therefore, as part of the protocol development, the following were tested: a) whether it was possible to identify the previously reported set of “canonical” RSNs with the Multiband sequence [81,88]; b) whether the Multiband sequence was effectively associated with a “better” signal relative to a Standard EPI sequence. For the first analysis MELODIC was used across the first 50 consecutive participants and was able to detect a set of RSNs that matched the “canonical” RSNs (Figure 4). For the second analysis a direct comparison was made between Multiband and Standard EPI sequences that had both been acquired on a subset (N =76) of participants. In order to be able to compare the two sequences with respect to spatial detail, spatial smoothing was not applied in this analysis. Additionally, an independent set of RSNs’ spatial maps, derived from data acquired for the Human Connectome Project (HCP), was used as a common template to extract time series and spatial maps from Multiband and Standard EPI sequence data using the dual regression approach [93,94]. HCP templates are of higher resolution and therefore contain more information compared with the standard template maps. Group maps were then obtained performing a one-sample *t*-test on the subjects’ spatial maps (output of the second stage of dual regression) for each component, calculating the corresponding z map and applying a mixture model correction to ensure comparable null distributions [95]. Figure 5 shows the quantile-quantile plots (Q-Q plots) of the group maps against the normal distribution. The group maps’ z-statistic distribution was expected to follow a normal distribution around zero (random noise – the null part of the spatial maps), while the tails should deviate from it according to the RSNs’ identified signal. Therefore our results suggest that Multiband sequence allows stronger RSN signal detection (higher z-values) with respect to Standard EPI sequence.

### Fluid attenuated inversion recovery (FLAIR)

**Figure 6:**
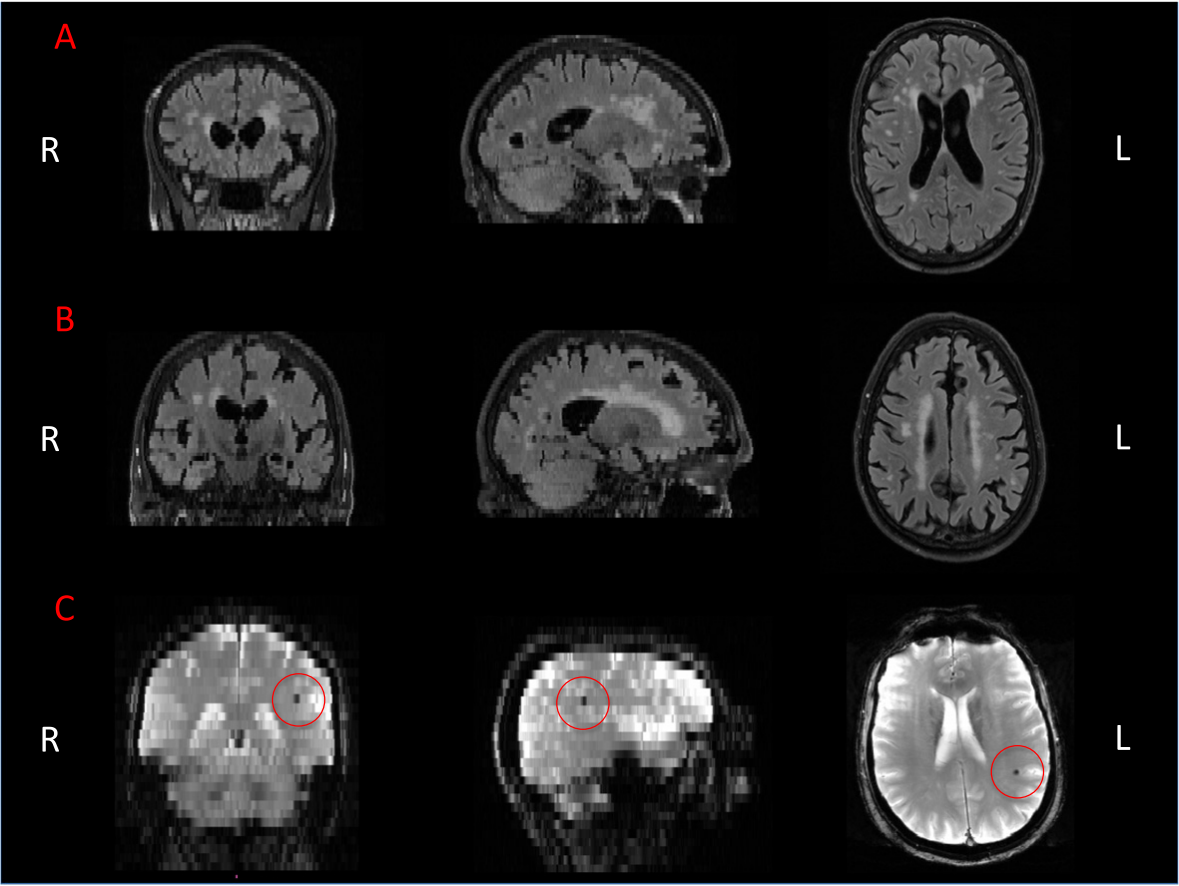
FLAIR and T2* images. (A) periventricular hyperintensities (PWH) and **(B)** deep white matter (DWM) lesions on FLAIR. **(C)** T2* image showing microbleed. R and L define the right and the left hemisphere, respectively.

This sequence is commonly used in clinical practice, for example to characterise periventricular lesions adjacent to the sulci, WM hyperintensities and WM lesions. Age-related increase in the number of hyperintensities and/or periventricular lesions has been widely reported [96,97]. Moreover, it has been shown that WM changes are associated with subtle structural and functional brain changes [98], and with decline in essential cognitive abilities in healthy elderly people [99]. Clinical ratings of FLAIR are performed by trained neuroscientists based on visual rating scores. WM lesions and/or hyperintensities are visually identified on FLAIR images by three independent raters who make assessments blind to demographic details. All axial slices of each subject are visually inspected. Peri-ventricular and deep WM hyperintensities are rated separately using a 4-point ordinal scale from 0–3; the sum of these two ratings lead to the total Fazekas score (an integer from 0–6) [100] (Figures 6A and B).

### T2* (T2 star)

The T2* sequence allows the identification of cerebral microbleeds, which reflect small deposits of the iron-storing protein hemosiderin in the brain and may be a sign of cerebral small-vessel disease [101,102]. Microbleeds can be found all over the brain and have been shown to be associated with neurological dysfunction and both clinical and cognitive impairment [103,104]. Their underlying mechanism is still under investigation but the deleterious effect, probably due to inflammatory effects, has been proved to affect neuron functionality and/or cortical cerebral activity [105]. All axial slices of T2* images are visually explored performed by trained neuroscientists and microbleeds identified (Figure 6C).

### Blood specimen protocol for the characterization of immune function

Following the MRI examination, blood samples (3 × 8 ml) and two buccal mucosal epithelial samples are taken from each participant. Blood samples are drawn using Vacutainer CPT tubes (Becton Dickinson) in a single venepuncture, inverted 8 times to mix anticoagulant additive with blood and processed within two hours of collection. Buccal mucosal epithelial cells are collected by nylon swabs (*microbiome sample*) and by twirling the brush against the epithelium and shaking the brush in RNAlater solution for 15 sec to remove the cells (*RNA sample*). CPT tubes are centrifuged in 1600 g for 30 min, allowing the separation of serum from white blood cells and from red blood cells. After the centrifugation, serum samples and peripheral blood mononuclear cells (PBMCs), i.e. T cells, B cells and NK cells are collected. PBMC cells are washed with phosphate-buffered saline (PBS), counted under microscope and cryopreserved using Cell Freezing Medium (5% DMSO/11%HSA in PBS). All samples are stored in −80°C for later analysis.

In order to investigate immune function in sample material, PBMC cells are stimulated *in vitro* with αCD3/αCD28 mAb and with TLR2/1 ligand Pam3CSK4 (Pam_3_Cys) and TLR4 ligand LPS in RPMI at 37°C in 5% CO_2._ Cells and supernatants are collected after 6 and 24 h of stimulation and stored for later analysis. RNA samples are investigated using RT-PCR or transcriptomics, while serum samples and cell supernatants are analysed with ELISA, Luminex or proteomics. Also, genomics/epigenomics and metabolomics techniques as well as advanced bioinformatics tools will be employed in the sample analysis.

### Hypotheses

We will examine the following overarching hypotheses in the sub-cohort recruited from WH II Phase 11:

(1) Early risk factors for cognitive dysfunction and late onset depression (such as cardio-vascular risk, see (i.) to (v.) below) will be associated with cerebral atrophy, reduced perfusion and impaired white matter integrity. Resulting impaired cognitive function (as measured by tests of episodic memory and executive function, see below) will be associated with specific brain volumes measured by advanced methods such as voxel based analysis and the automatic identification of specific structures, such as hippocampus, and also with reduced white matter integrity, quantified both globally [106], and homing in on fibres of interest (including measures of structural connectivity) [78];

(2) Resilience, measured in the sub-study directly [107] and by the absence of depression and cognitive impairment particularly in the presence of higher early risk (see above), is associated with high white matter integrity [108], and increased coherence of frontal regions in resting state networks (which provides a measure of functional connectivity) [109] or (compensatory) increased frontal BOLD signal in a memory encoding task [93]. The most important risk factors will be: (i) Antecedent vascular and metabolic risk trajectories and morbidity (adverse major blood lipids/apolipoproteins, hyperglycaemia and diabetes, adiposity, high blood pressure, smoking, chronic inflammation (C-reactive protein, interleukin 6), ECG abnormalities, angina, myocardial infarction, stroke; Framingham cardiovascular risk scores); (ii) low antecedent levels of physical and mental activity (measured by questionnaire [110-112]); (iii) baseline cognitive performance levels and up to 15-year gradients of memory and executive function decrement; (iv) history of depressed mood (3 to 8 repeat measurements using the CESD [12] and the GHQ [10], (v) genotype (APOE4 plus >48 k single nucleotide polymorphisms relevant to cardiovascular, metabolic and inflammatory syndromes [113]). The primary clinical hypotheses which we will address are: a) The quality of frontal compensatory activity will be affected by vascular risk, hypertension, including absence of protective factors, such as physical fitness, cardio-vascular and cerebrovascular prophylaxis (aspirin, statins, antihypertensives), by mental activity (“use it or lose it”), and by frontal lobe atrophy (as observed in treatment resistant depression); b) Time-trajectories derived from the WHII data set, e.g. of cognitive function or depression scores, and vascular risks-factor trajectories, will account for more of the diversity of outcome than singular measurements during any one of the previous follow-phases. This may allow imputations about the natural history of brain changes, but from clinician’s point of view, the least labour and time-intensive predictive test will be the most attractive. An important clinical task would, therefore, be to determine the minimum data set to predict outcome. c) Overall resilience, as supported by the absence of current and past affective or cognitive symptoms and good performance [78], can be modelled longitudinally from observations antecedent to medial temporal atrophy and frontal compensatory activity and structural integrity (high FA in DTI scan) as described in (a) and (b). In addition, we will address the following specific hypotheses which are of independent interest but will also support the primary findings: (d) Global cognitive performance depends on both hippocampal and frontal integrity/connectivity and (compensatory) frontal activity measured relatively higher frontal coherence within executive resting state networks; (e) Clinical impairment (outcome) appears if functional frontal compensation does not keep up with the degree of hippocampal and frontal atrophy observed; (f) First onset of depression after the age of 65 compared with no depression ever (outcome), matched for age, sex, education, and potential causal factors, such as vascular risk, is associated with reduced frontal/executive network structural and functional connectivity; (g) Clinical impairment may present as impaired cognition and/or as major depressive syndrome depending on the frontal networks affected; (h) Greater hippocampal atrophy will be observed with increasing age and APOE ϵ4 alleles; the absence of antidepressant medication; and among those with a family history of dementia.

## Discussion

The proposed programme of work will lead to the development and validation of methods and measures to integrate biological, physical, psychological, and socio-economic markers or indicators of health and wellbeing in later life, combining the Whitehall II repeat data from 1985 onwards and the psychiatric assessment in Phase 11 (2012–13), with detailed structural and functional imaging data collected in 2012–2016.

Our focus will be on understanding the development and impact of age-related conditions such as depression and cognitive decline, their implications for employment and work in later life, and specifically the neural mechanisms of compensation for cognitive decline and resilience to age-related stress by identifying the mechanisms of neural scaffolding, and the factors and mechanisms associated with successful ageing in the face of brain changes and risk factors. This hopefully will generate ways of protecting against age-related cognitive decline - an approach that is urgently needed, given the current limited progress in specifically preventing dementia.

The programme requires the close collaboration between the epidemiological and clinical team at UCL, the psychiatric and neuropsychological teams in the Oxford Department of Psychiatry and the expertise and resources of the neuroimaging centre at FMRIB. The research programme not only crosses boundaries within the MRC, but also involves engineering and physics components by virtue of its complex MRI acquisition and analysis protocol. The Whitehall II data-base includes extensive socio-economic data, which will allow testing of relevant hypotheses that cross over to risk factors employed in medical epidemiology, and cover the entire adult life course from early adulthood (age 35) to old age. The programme is relevant to biological mechanisms within the neurosciences, as the imaging methods employed will require further interpretation and possible adjunct projects to investigate the biological mechanism responsible for MRI abnormalities. There are of course other large-scale projects, including the UK Biobank and the Connectome Project. One aspect of specific added value in the Whitehall MRI substudy will be the availability of a fine-grained chronological clinical and life-style record with very detailed cognitive assessment and comprehensive MRI data covering structural and functional brain connectivity.

## Abbreviations

3 T: 3 Tesla (magnetic field strength); ACE-III: Addenbrooke’s cognitive examination 3rd revision; AD: Axial diffusivity; APOE: Apolipoprotein E gene/allele; B cells: B lymphocytes play a role in the humoral immunity of the adaptive immune system; BD: Becton Dickinson (company); BNT-60: Boston naming (60-item) test; BOLD: MRI blood oxygen level dependent; BPRS: Brief psychiatric rating scale; CANTAB RTI: Cambridge neuropsychological test automated battery reaction time; CES-D: Centre for epidemiological studies depression scale; CHAMPS: Community Healthy Activities Model Program for Seniors (Physical Activity Questionnaire for Older Adults); CHD: Coronary heart disease; CO2: Carbon dioxide; CPT: Cell preparation tube; CSF: Cerebrospinal fluid; CUREC: Central University Research Ethics Committee; DC: Digit coding test; DMSO: Dimethyl sulfoxide; DS: Digit span test; DSB: Digit span backwards; DSF: Digit span forwards; DSM-IV: Diagnostic and Statistical Manual 4th edition; DSS: Digit span rearranged in ascending sequence; DTI: MRI diffusion tensor imaging; dMRI: Diffusion weighted MRI; EEG: Electroencephalography; ELISA: Enzyme-linked immunosorbent assay; EPI: MRI echo planar imaging; FAST: FMRIB’s automated segmentation tool; FAST: Fractional anisotropy; FIRST: FMRIB’s model-based segmentation/registration tool; FIX: FMRIB’s ICA-based X-noiseifier; FLAIR: MRI fluid attenuated inversion recovery; FLASH: MRI fast low angle shot; FLIRT: FMRIB’s linear image registration tool; FMRIB Centre: Functional Magnetic Resonance Imaging of the Brain Centre (Oxford); FNIRT: FMRIB’s nonlinear image registration tool; FSL: FMRIB software library; FWHM: Full width at half maximum (spatial resolution); GHQ-30: General health questionnaire-30; GM: Brain grey matter; HAMD: Hamilton depression scale; HCP: Human connectome project; HRV: Heart rate variability; HSA: Human serum albumin; HVLT-R: Hopkins verbal learning test-revised; ICA: Independent component analysis; JSQ: Jenkins sleep questionnaire; LCE: Locus for causality exercise questionnaire; LOT-R: Life-orientation revised; LPS: Lipopolysaccharide (Endotoxin); LTE-Q: List of threatening experiences questionnaire; mAb: Monoclonal antibody; MD: Mean diffusivity; MDQ: Mood disorder questionnaire; MEMPR: Multi-echo MPRAGE; MGH: Massachusetts general hospital; MoCA: Montreal cognitive assessment; MPRAGE: MRI magnetization-prepared 180 degrees radio-frequency pulses and rapid gradient-echo; MRC: Medical Research Council (UK); MRI: Magnetic resonance imaging; MSD-IDREC: Medical Science Division Interdisciplinary Research Ethics Committee; NK cells: Natural killer cells (or NK cells) are a type of cytotoxic lymphocyte critical to the innate immune system; OCMR: Oxford Centre for Clinical Magnetic Resonance; Pam_3_Cys: (S)-(2,3-bis(palmitoyloxy)-(2RS)-propyl)-N-palmitoyl-(R)-Cys-(S)-Ser(S)-Lys4-OH trihydrochloride (synthetic lipopeptide, TLR2/1 agonist); PBMC: Peripheral blood mononuclear cells; PBS: Phosphate buffered saline; PE: Phase-encoding; PET: Positron emission tomography; PI: Principal investigator; PSQI: Pittsburgh sleep quality index; PSWQ: Penn state worry questionnaire ultra-brief version; Q-Q plots: Quantile-quantile plots; RCFT: Rey complex figure test; RD: Radial diffusivity; REC: Research Ethics Committee; RNA: Ribonucleic acid; RPMI: Roswell Park Memorial Institute; rfMRI: Resting-state functional MRI; RSN: Resting State Network; RT-PCR: Reverse transcription polymerase chain reaction; SCID-1: Structured clinical interview for DSM-IV-TR axis I disorders; SNR: Signal to noise ratio; STAI: State and trait anxiety inventory; T cells: T lymphocytes play a central role in cell-mediated immunity; T1: MRI spin–lattice relaxation time; T2*: MRI measure of the loss of coherence in an ensemble of spins that includes all interactions (including static dephasing); TLR: Toll-like receptor; TMT: Trail making test; TOPF: Test of premorbid functioning; UCL: University College London; WAIS-IV: Wechsler Adult Intelligence Scale - fourth edition; WHII: The Whitehall II study; WHII Phase 11: Whitehall Two Phase 11 (2011–2012); WM: Brain white matter; Y-BOCS: Yale-Brown obsessive compulsive scale; YMRS: Young mania rating scale.

## Competing interests

*Financial competing interests*: NF, EZs, RH, CES, AM, CLA, AT, VV, EJB, MJS, EA, SM, KU, JX, EY, JA, JB, SC, LG, ATH, MJ, KLM, SNS, NLV, SMS, JRG, AS-M, CEM, MK declare no financial competing interest. GR-K is director of R&D in AIG, with potential financial competing interests. KPE has received consultation fees from Eli Lilly in connection with Amyvid™. *Non-financial competing interests*: NF, EZs, RH, CES, AM, CLA, AT, VV, EJB, MJS, EA, SM, KU, JX, EY, JA, JB, SC, LG, ATH, MJ, KLM, GS-K, SNS, NLV, SMS, JRG, AS-M, CEM, MK, and KPE declare no non-financial competing interests.

## Authors’ contributions

NF wrote part of the first draft, planned the imaging protocol and performed some of the analyses. EZs wrote part of the first draft, planned the imaging, clinical and neuropsychological protocol, as well as the data management, and performed some of the analyses. RH wrote part of the first draft, planned the immunology protocol and performed some of the analyses. CES planned the data management, as well as the imaging, clinical and neuropsychological protocol, and critically read the manuscript. AM contributed to the data management, as well as some of the clinical and neuropsychological protocol, and critically read the manuscript. CLA contributed to planning the study and critically read the manuscript. AT and VV reviewed the clinical and neuropsychological protocol, and critically read the manuscript. EJB and MJS contributed to the data analysis plan and critically read the manuscript. EA, SM, KU, JX and EY led the development of multiband image acquisition sequences, and critically read the manuscript. JB, KLM, LG, NLV, RSK and SC set up the multiband sequence development and analysis pipeline and critically read the manuscript. JA, MJ and SNS assisted with the Diffusion MRI acquisition protocol, set up the data pre-processing pipeline and critically read the manuscript. ATH set up the structural sequence development and analysis pipeline and critically read the manuscript. SMS planned the study, contributed to the application for funding and critically revised the draft of this paper. JRG planned the study, contributed to the application for funding and critically revised the draft of this paper. ASM planned the study, contributed to the application for funding and critically revised the draft of this paper. CEM planned the study, contributed to the application for funding and critically revised drafts of this paper. MK planned the study, contributed to the application for funding and critically revised the draft of this paper. KPE (PI) planned the study, contributed to the application for funding, managed the study, and critically revised drafts of this paper. All Authors read and approved the final manuscript.

## Pre-publication history

The pre-publication history for this paper can be accessed here:

http://www.biomedcentral.com/1471-244X/14/159/prepub
